# Screening for Peripheral Artery Disease Using an Automated Four-Limb Blood Pressure Monitor Equipped with Toe–Brachial Index Measurement

**DOI:** 10.3390/jcm12206539

**Published:** 2023-10-15

**Authors:** Krisztina Fendrik, Katalin Biró, Dóra Endrei, Katalin Koltai, Barbara Sándor, Kálmán Tóth, Gábor Késmárky

**Affiliations:** 1 Division of Angiology, 1st Department of Medicine, Clinical Centre University of Pécs, University of Pécs Medical School, Ifjúság útja 13, H-7624 Pécs, Hungary; biro.katalin@pte.hu (K.B.); endrei.dora@pte.hu (D.E.); koltai.katalin@pte.hu (K.K.); kesmarky.gabor@pte.hu (G.K.); 2 Division of Cardiology, 1st Department of Medicine, Clinical Centre University of Pécs, University of Pécs Medical School, Ifjúság útja 13, H-7624 Pécs, Hungary; sandor.barbara@pte.hu (B.S.); toth.kalman@pte.hu (K.T.)

**Keywords:** peripheral artery disease, screening, ankle–brachial index, toe–brachial index, oscillometry, photoplethysmography

## Abstract

Toe–brachial index (TBI) measurement helps to detect peripheral artery disease (PAD) in patients with incompressible ankle arteries due to medial arterial calcification, which is most frequently associated with diabetes. We aimed to evaluate how an automated four-limb blood pressure monitor equipped with TBI measurement could contribute to PAD screening. In 117 patients (mean age 63.2 ± 12.8 years), ankle–brachial index (ABI) measurement was performed using the Doppler-method and the MESI mTablet. TBI was obtained via photoplethysmography (MESI mTablet, SysToe) and a laser Doppler fluxmeter (PeriFlux 5000). Lower limb PAD lesions were evaluated based on vascular imaging. A significant correlation was found between Doppler and MESI ankle–brachial index values (r = 0.672), which was stronger in non-diabetic (r = 0.744) than in diabetic (r = 0.562) patients. At an ABI cut-off of 0.9, Doppler (AUC = 0.888) showed a sensitivity/specificity of 67.1%/97.4%, MESI (AUC 0.891) exhibited a sensitivity/specificity of 57.0%/100%; at a cut-off of 1.0, MESI demonstrated a sensitivity/specificity of 74.7%/94.8%. The TBI values measured using the three devices did not differ significantly (*p* = 0.33). At a TBI cut-off of 0.7, MESI (AUC = 0.909) revealed a sensitivity/specificity of 92.1%/67.5%. Combining MESI ABI and TBI measurements recognised 92.4% of PAD limbs. Using an ABI cut-off level of 1.0 and sequential TBI measurement increases the sensitivity of the device in detecting PAD. The precise interpretation of the obtained results requires some expertise.

## 1. Introduction

Peripheral artery disease (PAD) is a progressive atherosclerotic disorder of the abdominal aorta and the arteries of the upper and lower extremities, which, if left untreated, leads to stenosis or occlusion of the affected vessels. It is the third leading cause of atherosclerotic mortality, following coronary heart disease and stroke [1]. PAD is a growing health problem; it is estimated that more than 230 million people suffer from PAD worldwide. The prevalence increases with age, and it can affect one in six people older than 80 years [2]. The spectrum of symptoms is wide. Intermittent claudication (i.e., leg muscle pain or fatigue brought on by exercise and relieved on rest), which is considered a typical symptom of the disease, occurs only in 5–10% of patients [3], while approximately 20–50% have no exertional leg symptoms and 40–60% have atypical leg symptoms [4]. Ischaemic leg symptoms are often wrongly considered a consequence of musculoskeletal diseases, mainly in elderly patients.

The existence of PAD is a strong predictor of other atherosclerotic diseases; it is associated with at least comparable risk of all-cause and cardiovascular (CV) mortality to cardio- and cerebrovascular diseases [5]. Despite its high prevalence, association with adverse clinical outcomes, and high impact on quality of life and physical activity, PAD very often remains unrecognised.

The non-invasive test of choice after clinical examination is the Doppler-assisted measurement of the ankle–brachial index (ABI) [3,6,7,8,9]. This test is cost-effective and is widely available, also in primary care. However, its wide use is limited by time and personnel constraints since its correct implementation requires expertise [10,11,12].

To overcome the limitations of the Doppler method, automated, oscillometric devices have been developed in recent years, which are specially designed for ABI measurement. The simultaneous and automated blood pressure measurement on all four limbs helps to eliminate measurement inaccuracies due to blood pressure fluctuations and lack of experience by the examiner and reduces the time required to perform the examination [13]. Studies comparing oscillometric devices with the Doppler method are partly controversial [14]. However, despite the limitations of these devices, including low measurement accuracy in low ABI ranges [15,16,17,18] and a tendency to slightly overestimate ABI values compared to the Doppler measurement [19], a recent meta-analysis concluded that oscillometric devices have an acceptable diagnostic accuracy and feasibility, and the forementioned advantages may make these devices useful, especially in mass screening programmes for PAD [13]. To increase the sensitivity of automated equipments in detecting PAD, manufacturers have equipped these devices with various additional functions in recent years, like measurement of estimated carotid–femoral pulse wave velocity (ecfPWV) or toe–brachial index (TBI). The latter helps to overcome the constraints of resting ABI measurement in patients with incompressible ankle arteries due to medial arterial calcification (MAC), which is most frequently associated with diabetes, chronic kidney disease or advanced age [20,21]. These conditions can result in falsely elevated ABI values [22,23]. If no further investigations are carried out, the diagnosis of PAD can fail in these patients. Since MAC usually does not affect the toe arteries [24], current guidelines recommend alternative tests, such as TBI measurement to detect PAD in patients with incompressible ankle arteries or in case of a high ABI (>1.4) [3,8,9]. Toe pressure measurement is usually carried out by measuring systolic blood pressure on the hallux or, in its absence, on the second toe using photoplethysmographic (PPG) or laser Doppler (LD) principle [25,26]. Despite the clinical importance of toe pressure measurement, it is an almost neglected screening method in outpatient settings.

The aim of this study was to test an automated, wireless device which combines the benefits of oscillometric ABI measurement and PPG TBI measurement (MESI mTablet, MESI Ltd., Ljubljana, Slovenia). We aimed to investigate the diagnostic accuracy of ABI and TBI measurements, taking vascular imaging techniques as a reference. The TBI measurement function was compared to the LD method (PeriFlux 5000, Perimed AB, Järfälla, Sweden) and another portable device operating on the PPG principle (SysToe, Atys Medical, Soucieu en Jarrest, France). We aimed to analyse how the device can contribute to PAD screening.

## 2. Materials and Methods

### 2.1. Study Design

Our study was performed with an inclusion of 117 patients, aged from 27 to 83 years (mean age 63.2 ± 12.8 years). The inclusion criteria were ≥18 years of age and a signed written informed consent. Patients were recruited from April to November 2022 in the outpatient clinic and in the ward of the Division of Angiology at the University of Pécs Clinical Centre. Patients were screened prospectively and consecutively. Among the included subjects, 18 volunteers belonged to the control group. The "Control” group (mean age 60.4 ± 11.9 years) involved age-matched (±5 years tolerance compared to all patients) non-smoking persons without diabetes or any CV diseases, except for uncomplicated, medically properly treated essential hypertension. Twelve patients belonging to the “Other CV” group suffered from non-atherosclerotic CV diseases, mainly from venous thromboembolic diseases. According to the ”2021 ESC Guidelines on cardiovascular disease prevention in clinical practice” [27], 28 subjects were classified as “High CV Risk” and another 28 subjects as “Very High CV Risk” patients. Thirty-one patients with pre-known PAD were also involved (“Confirmed PAD”). PAD was defined as at least moderate stenosis of the arteries of the lower extremities (luminal stenosis greater than 50% of the lumen) [28].

### 2.2. Methods

Medical history, co-morbidities, CV risk factors and concomitant medication were obtained from every patient. The instrumental measurements were performed by the same experienced operator following the same examination sequence for all study patients. Participants were asked to avoid alcohol, caffeine and smoking at least one hour before the examination. Before starting any investigation, participants were acclimatized to controlled room temperature (22–24 °C) for at least 5 min in the lying position. All measurements were performed in the supine position.

#### 2.2.1. ABI Measurement

##### Doppler-Assisted ABI Measurement

The examination was performed using a manual sphygmomanometer and a hand-held continuous-wave Doppler ultrasound device (Bidop ES-100V3, Hadeco, Inc., Kawasaki, Japan) equipped with an 8 MHz probe. Systolic blood pressure of the posterior tibial (PTA) and dorsal pedal artery (DPA) on both lower limbs, as well as of the brachial artery on both arms, was measured following the same sequence (right arm—right leg—left leg—left arm). The cuffs were positioned approximately 1 cm above the ankle joints and above the elbow joints with parallel wrapping. ABI was calculated in two different ways—for the Doppler ABI, the higher systolic blood pressure, while for the modified Doppler ABI, the lower systolic blood pressure, of the PTA and DPA of each leg was divided by the higher systolic blood pressure between both arms.

##### ABI Measurement with an Automated, Oscillometric Blood Pressure Monitor

Measurements were performed using a wireless four-limb blood pressure monitor, MESI mTablet (MESI Ltd., Ljubljana, Slovenia). Various modules (blood pressure, ABI and TBI measurements, 12-lead electrocardiogram, spirometry, pulse oxymetry) can be connected to the central tablet via Bluetooth. ABI measurements were conducted using the dedicated software installed on the tablet [29].

The measurements were carried out using the oscillometric principle. After placing the right-sized, wireless, colour-coded cuffs on both upper arms (1–2 cm above the elbow joint) and ankles (2–3 cm above the inner ankle), the measurement could be started using the application on the tablet. In the first step, the arm cuffs were inflated, and the arm with the higher systolic pressure was selected based on the Smartarm^TM^ algorithm. In the second step, the two ankle cuffs and the selected arm cuff were inflated simultaneously. In addition to the calculated both-sided ABI value, oscillation graphs and pulse waves were also displayed. By analysing the morphology of the pulse waves, the PADsense^TM^ algorithm raises the possibility of severe PAD (usually ABI ≤ 0.5) or incompressible arteries due to mediasclerosis. In this case, ABI is not calculated, and the device provides a text information (“Possibility of severe PAD or incompressible arteries” or “Abnormally weak pulse”) [30].

An ABI value ≤ 0.9 was considered abnormal. Values > 1.4 were considered to indicate mediasclerosis.

#### 2.2.2. TBI Measurement

Systolic toe pressure was measured using three different devices, first with the MESI mTablet based on PPG, followed by LD flowmetry and, finally, with another portable device operating also on the PPG principle.

##### TBI Measurement Using the MESI mTablet

The colour-coded, appropriately sized toe cuffs were wrapped sufficiently tightly around the first toe of both lower limbs. The PPG sensor was fixed above the cuffs on the plantar surface of the first toe. The two toe cuffs and the two arm cuffs were simultaneously inflated to a suprasystolic value relative to the upper arm blood pressure, and then deflated simultaneously at a specific rate. The reappearance of the first normal-amplitude pulse wave on the toes were detected using the FirstWave^TM^ algorithm. The pressure measured at this time corresponded to the systolic toe pressure. If not accepted by the examiner, the curve was adjusted manually. TBI values were calculated by dividing the toe pressure values with the higher arm pressure and were displayed on the tablet [31].

##### Toe Pressure Measurement Using LD Flowmetry

This measurement was carried out using a PeriFlux System 5000 device (Perimed AB, Järfälla, Sweden) based on the linear deflation method, and the analysis was performed using a dedicated software (PeriSoft v2.50). The laser probe was positioned on the plantar surface of the distal phalanx of the first toe using a double-sided adhesive tape. The perfusion curve was displayed by the software. An appropriate-sized toe cuff was wrapped around the toe under the probe. The inflation of the cuff was started by pressing the pump connected to the device, and after reaching 200 mmHg, the pressure was automatically decreased in small steps. The reappearance of the perfusion curve corresponded with the systolic toe pressure, and this could be determined using a special feature of the software [32].

##### Toe Pressure Measurement by the SysToe PPG Device

The portable, battery-operated device (SysToe, Soucieu en Jarrest, Atys Medical, France) also carried out toe pressure measurement using the infrared PPG principle. Changes in the skin blood volume were detected using a photoelectric sensor. The occlusion cuff was wrapped around the proximal phalanx of the first toe, and the cuff with the built-in sensor was placed above it. The perfusion curves were displayed on the device’s screen. After starting the measurement, the occlusion cuff was inflated to a pressure of 300 mmHg, and then deflated automatically at a specified rate. A steeply rising curve indicated the return of blood flow and, thereby, the systolic toe pressure [33,34].

TBI for each lower limb was determined by dividing the systolic toe pressure with the higher systolic arm pressure measured using the Doppler method. A TBI value ≤0.7 was considered abnormal.

#### 2.2.3. Vascular Imaging

All patients underwent a vascular imaging technique; this included colour-coded duplex ultrasound examination in 92 cases, digital subtractional angiography (DSA) in 20 cases and CT angiography in 1 case. In 4 cases of pre-known, chronic, non-intervenable PAD, previous DSA documentation was available and, therefore, no repeated examination was performed. PAD was defined by the presence of at least one significant (at least 50%) stenosis of the lower limb arteries.

##### Colour-Coded Duplex Ultrasound of the Lower Extremity Arteries

These examinations were performed using a GE Vivid S60N v202CH (SN 003732S60) device equipped with a 10 MHz linear probe by a single investigator. The distal part of the common femoral artery, the proximal and middle thirds of the superficial femoral artery, and the popliteal, peroneal and distal parts of the PTA and DPA were analysed, respectively, by obtaining two-dimensional images and pulsed Doppler spectral waveforms with the analysis of the peak systolic velocity (PSV). Due to abdominal adiposity or meteorism, in several patients, it was not possible to obtain a flow profile directly from the iliac arteries; thus, only the analysis of the Doppler waveforms derived from the common femoral arteries could be used to indirectly refer to them. Atherosclerotic plaques were defined as an intima–media thickness exceeding the one of the neighbouring sites by at least 50% [35]. Stenoses were evaluated using the PSV ratio (ratio of PSV at stenosis to the PSV measured directly proximal to the stenosis) and were considered significant when >2 [36]. Due to the limited evaluability of duplex ultrasound on the calf, the criteria of the Hodgkiss–Harlow classification were also used to define a stenosis exceeding 50% [37].

##### Digital Subtraction Angiography

Patients with an at least Fontaine stage IIb PAD underwent a DSA with sequential endovascular intervention. The examination was performed in the Department of Medical Imaging at the University of Pécs Clinical Centre with a Siemens Axiom Artis dTA (SN 55371) device. The catheter was inserted via transfemoral or transbrachial approach, followed by the intra-arterial administration of the contrast agent. The obtained images of anteroposterior sequential views of the lower abdomen, pelvis and lower extremities were evaluated by experienced angiographers. The visual stenosis estimate was performed via comparison with the normal surrounding arterial segments.

##### CT Angiography

This examination was performed in the Department of Medical Imaging at the University of Pécs Clinical Centre using a GE Medical Systems Revolution CT (SN REVVX2000052CN) device, after the intravenous administration of the contrast agent. The multidimensional reconstruction of the abdominal, pelvic and lower limb arteries was evaluated by an experienced radiologist.

### 2.3. Statistical Analysis

Statistical analysis was performed using Statistical Product and Service Solutions (SPSS) statistical software, version 28.0.0.0 (SPSS Inc., Chicago, IL USA). Continuous variables were expressed as mean ± standard deviation (SD). The between- and within-group analyses of continuous variables were performed using one-way ANOVA (the ABI and TBI values obtained using various methods were used as the dependent variable, and the patient groups as the independent variable). Homogeneity of variances was evaluated based on Levene’s test. For inhomogeneity of variances, Welch’s ANOVA statistics and Tamhane’s post hoc test were performed. The means of the automated and manually adjusted TBI values were compared using paired samples *t*-test. The association between the Doppler-assisted and automated MESI ABI measurements was analysed based on Pearson product-moment correlation. The intermodality agreement of the Doppler and MESI ABI measurements, as well as the LD and MESI TBI readings, was evaluated using the Bland–Altman analysis [38]. A linear regression analysis was performed to detect proportional bias. The diagnostic efficacy of the different measurement techniques was compared using receiver operating characteristic (ROC) curve analysis, where we used ABI and TBI values as the test variable, and the presence or absence of PAD as the state variable. The area under curve (AUC) value was used to estimate the accuracy of the various diagnostic tests. The optimal cut-off value for each method was calculated using Youden’s J statistic based on the “sensitivity + specificity −1” equation. The cut-off value belonging to the highest Youden’s J index was selected.

Parameters were analysed with the cooperation of two authors.

A *p*-value < 0.05 was considered to show statistical significance.

## 3. Results

The demographics and baseline characteristics of the study sample are summarised in Table 1.

### 3.1. ABI Measurement

In total, 233 lower limbs of 117 patients could be examined, due to a previous unilateral major amputation of one subject. The MESI mTablet showed numerical ABI data in 210 cases. In another 23 cases, a text signal of “Possibility of severe PAD or incompressible arteries” was displayed. The ratio of patients who lacked numerical data was 14.6% in the subgroup of diabetic patients (*n* = 41, 12 text data of 82 readings) and 7.6% in non-diabetic patients (*n* = 76, 11 text data of 151 ABI measurements). By comparing these readings with the results of the vascular imaging, it could be ascertained that 100% of these limbs could be diagnosed with PAD lesions. However, these 23 measurements had to be excluded from further statistical analysis. It was not possible to determine an ABI using the Doppler method due to incompressible arteries in six cases (four limbs in diabetic patients and two limbs in non-diabetic patients). Out of the analysed 204 limbs, the mean Doppler ABI was 1.04 ± 0.20, the mean modified Doppler ABI was 0.96 ± 0.22 and the mean MESI ABI was 1.10 ± 0.15. The Doppler, modified Doppler and MESI ABI values in the various subgroups are summarised in Table 2.

The Welch’s ANOVA test revealed significant differences among the Doppler, modified Doppler and MESI ABI values between the five subgroups—for Doppler ABI, F(4,97.508) = 31.581, *p* < 0.001; for modified Doppler ABI, F(4,96.447) = 37.743, *p* < 0.001; and for MESI ABI, F(4,89.128) = 28.150, *p* < 0.001.

The mean Doppler, modified Doppler and MESI ABI values of the Confirmed PAD patients significantly differed from the corresponding values of all other subgroups (*p* < 0.001 for all comparisons). We revealed a significant difference between the mean Doppler ABI values of the Control group and the Very High CV Risk group (*p* < 0.001). The mean modified Doppler ABI of the Control group differed significantly from the values of the High (*p* = 0.003) and Very High (*p* < 0.001) CV Risk groups. The mean MESI ABI of the Control group differed significantly from the values of all other groups (*p* = 0.005 for the Control vs. Other CV, *p* < 0.001 for the Control vs. High CV Risk and Control vs. Very High CV Risk comparisons).

The Pearson correlation analysis revealed a strong, significant correlation between the Doppler-assisted and oscillometric MESI ABI readings (r = 0.672, *p* < 0.001). The correlation was stronger in non-diabetic (r = 0.744, *p* < 0.001) than in diabetic (r = 0.562, *p* < 0.001) patients.

**Figure 1:**
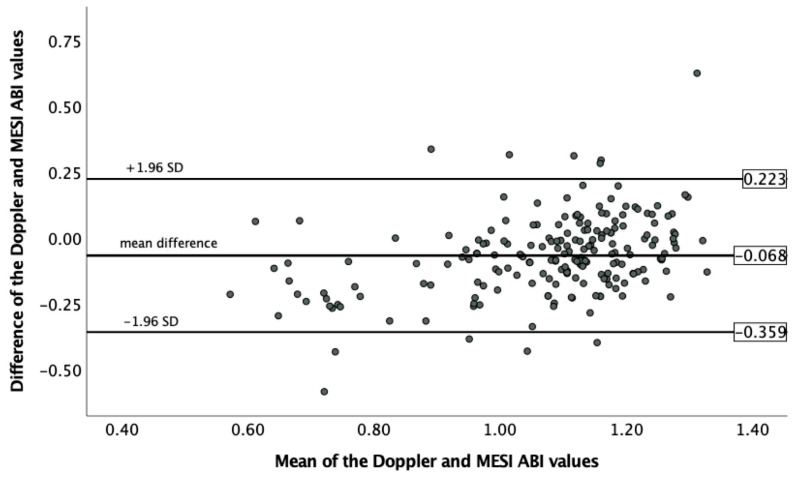
Analysis of the intermodality agreement between the Doppler and MESI ABI measurements using the Bland–Altman method. The mean of the corresponding Doppler and MESI ABI values on the ‘*x*’ axis is plotted against the difference of the corresponding Doppler and MESI ABI values on the ‘*y*’ axis. The horizontal lines represent the mean difference with the limits of agreement (ABI, ankle–brachial index; SD, standard deviation).

The Bland–Altman plot (Figure 1) displays a mean difference of -0.068 between the Doppler and MESI ABI measurements, with the limits of agreement of 0.223 and −0.359. The linear regression analysis indicated the presence of proportional bias (R^2^ = 0.149, F(1,202) = 35.238, *p* < 0.001).

**Figure 2:**
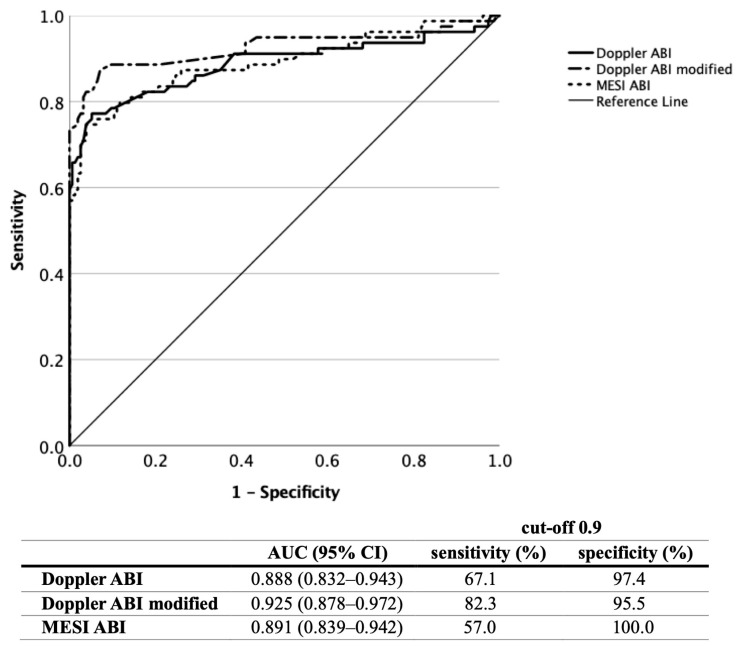
Diagnostic efficacy of the Doppler, modified Doppler and MESI ABI measurements based on ROC curve analysis in all patients (ABI, ankle–brachial index; ROC, receiver operating characteristic; AUC, area under the curve).

The diagnostic efficacy of the three different ABI readings in recognising PAD with reference to the vascular imaging was analysed based on ROC curves (Figure 2).

For the cut-off value of 0.9 ABI, the Doppler ABI [AUC = 0.888, (95% CI 0.832–0.943), *p* < 0.001] showed a sensitivity/specificity of 67.1%/97.4%, the modified Doppler ABI [AUC = 0.925, (95%CI 0.878–0.972), *p* < 0.001] showed 82.3%/95.5%, and the MESI ABI [AUC = 0.891, (95% CI 0.839–0.942)] showed 57.0%/100%.

For the cut-off value of 1.0, the MESI ABI showed a sensitivity of 74.7% and a specificity of 94.8%. The optimal cut-off value was calculated as 0.99 for the oscillometric MESI ABI determination.

**Figure 3:**
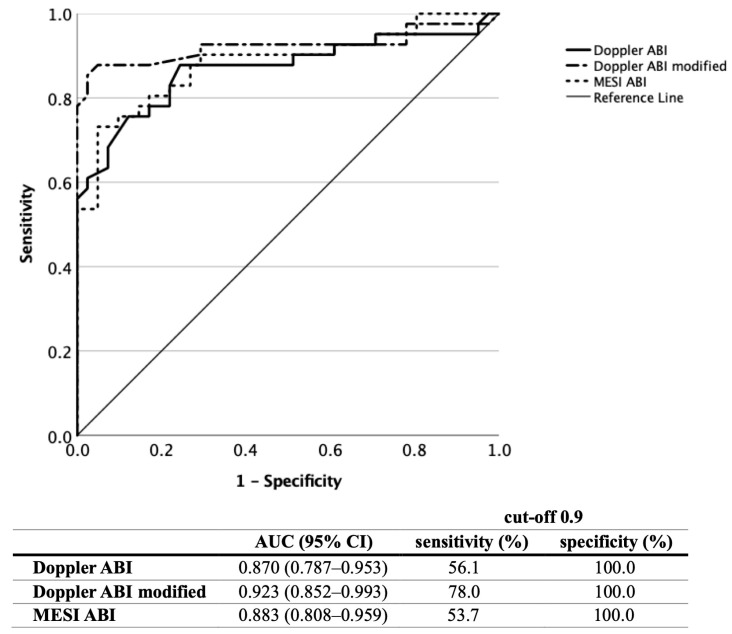
Diagnostic efficacy of the Doppler, modified Doppler and MESI ABI measurements based on ROC curve analysis in diabetic patients (ABI, ankle–brachial index; ROC, receiver operating characteristic; AUC, area under the curve).

In diabetic patients [Figure 3] (*n* = 41), a comparable diagnostic efficacy was found, with an AUC value of 0.870 for the Doppler ABI (95% CI 0.787–0.953) and 0.883 (95% CI 0.808–0.959) for the MESI ABI (*p* < 0.001 for both), while the modified Doppler ABI was linked with an AUC value of 0.923 (95% CI 0.852–0.993, *p* < 0.001). At an ABI cut-off value of 0.9, the Doppler method had a sensitivity/specificity of 56.1%/100%, the modified Doppler ABI had had a sensitivity/specificity of 78.0%/100%, and the MESI ABI had a sensitivity/specificity of 53.7%/100%. Raising the oscillometric ABI cut-off level to 1.0 resulted in a sensitivity of 73.2% and a specificity of 92.7%.

**Figure 4:**
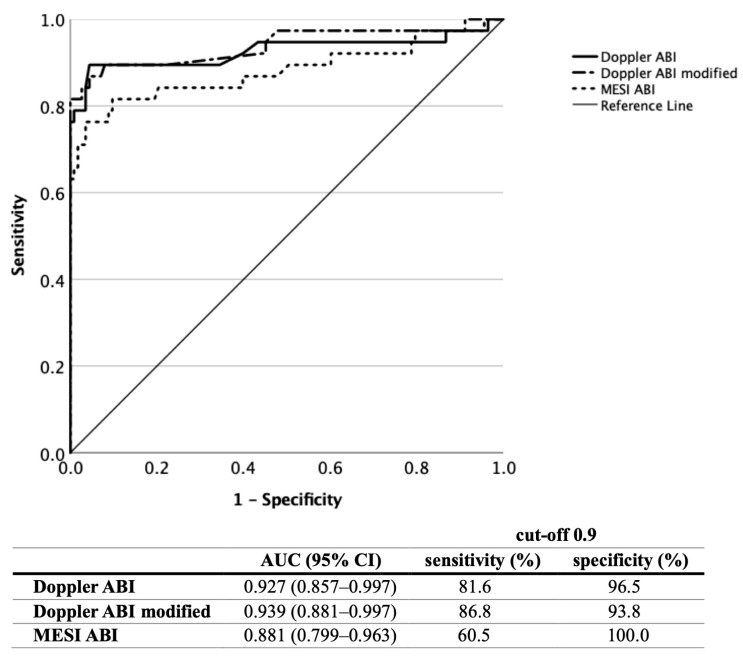
Diagnostic efficacy of the Doppler, modified Doppler and MESI ABI measurements based on ROC curve analysis in non-diabetic patients (ABI, ankle–brachial index; ROC, receiver operating characteristic; AUC, area under the curve).

In non-diabetic subjects [Figure 4] (*n* = 76), the Doppler ABI assessment revealed an AUC value of 0.927 (95% CI 0.857–0.997), while the modified ABI revealed a value of 0.939 (95% CI 0.881–0.997). The MESI ABI was the less efficient, with an AUC value of 0.881 (95% CI 0.799–0.963, *p* < 0.001 for all three analyses). At an ABI cut-off value of 0.9, the Doppler ABI showed a sensitivity/specificity of 81.6%/96.5%, the modified Doppler ABI showed a sensitivity/specificity of 86.8%/93.8%, and the MESI ABI showed a sensitivity/specificity of 60.5%/100%. Taking the oscillometric ABI cut-off level of 1.0 raised the sensitivity of the MESI ABI to 76.3% with a specificity of 95.8%.

### 3.2. TBI Measurement

Toe pressure measurement was performed using the three different devices on 230 lower limbs. In one case, toe pressure could not be obtained due to major amputation; in two cases, it was due to minor amputations; and in one case, it was due to toe gangrene. The toe pressure values of the automated MESI TBI measurement had to be corrected manually in most cases due to movement artifacts, which were recognised as a reappearance of the perfusion curve by the device. The automatically measured toe pressure values were noticed in 165 cases. However, the corrected values compared to the manually adjusted values showed no statistically significant difference (mean of the automated measurement 90.99 ± 40.88 mmHg, mean of the corrected values 86.81 ± 37.36 mmHg; paired samples *t*-test, t(164) = −1.087, *p* = 0.279).

The mean TBI was 0.66 ± 0.24 when measured using PeriFlux LD, 0.68 ± 0.23 when measured using SysToe and 0.65 ± 0.29 when measured using MESI, respectively.

Welch’s ANOVA revealed no significant differences between the three measurement techniques [F(2,454.056) = 1.102, *p* = 0.333). Tamhane’s post hoc test also showed no significant differences between the PeriFlux LD and SysToe measurements (*p* = 0.778), between the PeriFlux LD and MESI measurements (*p* = 0.868) and between the SysToe and MESI (*p* = 0.372) measurements. The mean TBI values of the three different measurement techniques divided into various subgroups are demonstrated in Table 3.

When comparing the data of the five subgroups, the mean values of all three TBI measurement techniques showed significant differences (*p* < 0.001 for all three comparisons).

All three types of mean TBI values differed significantly between the Confirmed PAD group and all other subgroups (*p* < 0.001 for all comparisons) and between the Control group and Very High CV Risk group (*p* = 0.01 for MESI TBI, *p* < 0.001 for PeriFlux LD and SysToe TBI).

**Figure 5:**
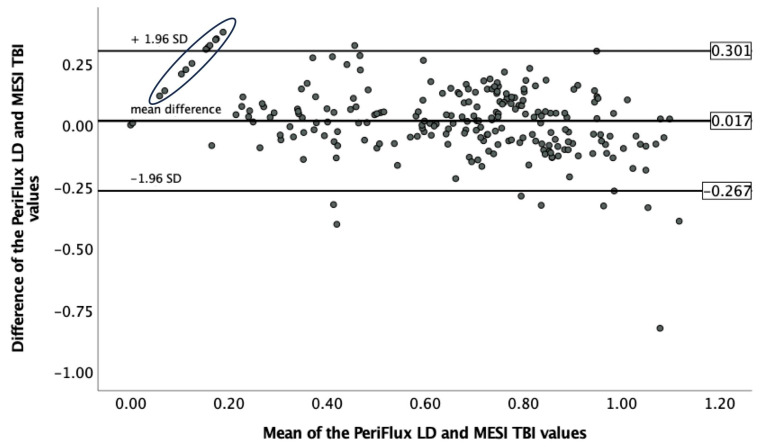
Analysis of the intermodality agreement between the PeriFlux LD and MESI TBI measurements using the Bland–Altman method. The mean of the corresponding PeriFlux LD and MESI TBI values on the ‘*x*’ axis is plotted against the difference of the corresponding PeriFlux LD and MESI TBI values on the ‘*y*’ axis. The horizontal lines represent the mean difference with the limits of agreement. The circled section demonstrates 11 cases in which the PeriFlux LD measured a numerical value, but the MESI device did not detect a pulse wave on the affected toe. (LD, laser Doppler; TBI, toe–brachial index; SD, standard deviation).

The Bland–Altman analysis showed a mean difference of 0.017 between the TBI assessment using the PeriFlux LD and the MESI measurements. The limits of agreement covered a range from −0.267 to 0.301. The circled section on Figure 5 demonstrates 11 cases in which the PeriFlux LD measured a numerical value, but the MESI device did not detect a pulse wave on the affected toe.

**Figure 6:**
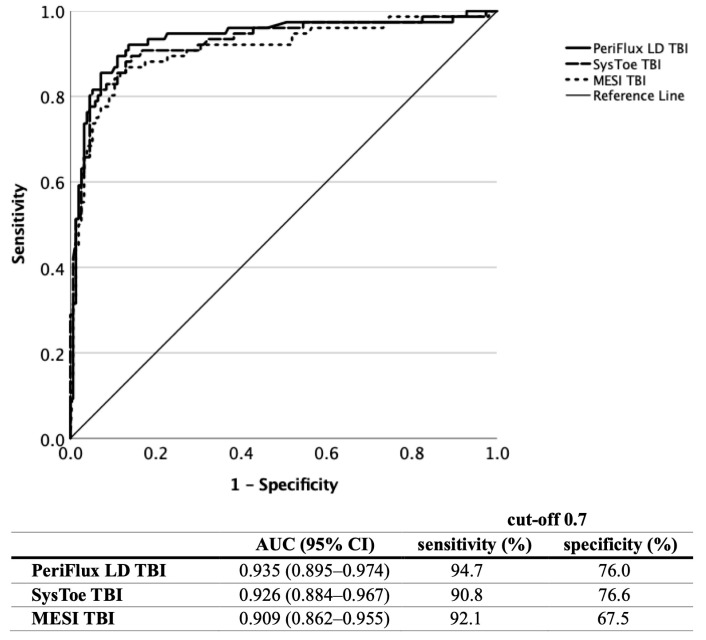
Comparison of the diagnostic efficacy of the PeriFlux LD, SysToe and MESI TBI measurements based on ROC curve analysis (LD, laser Doppler; TBI, toe–brachial index; ROC, receiver operating characteristic; AUC, area under the curve).

The ROC curve analysis of all three measurement techniques (Figure 6) revealed an excellent diagnostic efficacy, with the PeriFlux LD TBI measurement showing an AUC of 0.935 (95% CI 0.895–0.974), SysToe showing a value of 0.926 (95% CI 0.884–0.967) and MESI showing a value of 0.909 (95% CI 0.862–0.955), with a significance of *p* < 0.001 for all analyses.

PeriFlux LD showed a sensitivity/specificity of 94.7%/76.0%, SysToe showed a sensitivity/specificity of 90.8%/76.6%, and MESI showed a sensitivity/specificity of 92.1%/67.5% at a cut-off value of 0.7. The optimal cut-off value for the MESI TBI assessment was calculated to be 0.61.

### 3.3. Screening Using the Various Methods

The number of patients screened as positive by the different ABI and TBI measurements is demonstrated in Table 4. For the purpose of screening, the text message of PAD as displayed by the MESI ABI measurement and Doppler ABI values indicating mediasclerosis were also considered to be positive.

ABI values ≤ 0.9 or >1.4 and TBI values ≤ 0.7 were considered abnormal.

**Table 4:** Patients screened as positive using the different measurement techniques when compared with the results of vascular imaging (ABI, ankle–brachial index; LD, laser Doppler; TBI, toe–brachial index; PAD peripheral artery disease; CV, cardiovascular).

							Vascular Imaging	
	Doppler ABI	Doppler ABI Modified	MESI ABI	PeriFlux LD TBI	SysToe TBI	MESI TBI	PAD	Atherosclerosis
Control (*n* = 18)	0	0	0	1	0	3	0 (0.0%)	2 (11.1%)
Other CV (*n* = 12)	1	1	1	4	4	4	1 (8.3%)	1 (8.3%)
High CV risk (*n* = 28)	3	3	2	12	13	13	4 (14.3%)	19 (67.9%)
Very high CV risk (*n* = 28)	8	11	3	17	16	17	8 (28.6%)	26 (92.9%)

We also analysed how many of the 79 lower limbs of 44 patients affected by PAD (pre-known and newly diagnosed) could be recognised by the various measurement techniques. The Doppler ABI calculation gave abnormal results in 56 cases (70.9%), while the modified Doppler ABI calculation gave abnormal results in 66 (83.5%) cases. The discrepancies between these data and the data shown in the ROC curves resulted from ABI values indicating mediasclerosis, which were also considered abnormal. The MESI ABI recognised 45 limbs (57.0%) with text or numerical data at an ABI cut-off level of 0.9 and 59 limbs (74.7%) at an ABI cut-off level of 1.0, respectively.

TBI was obtainable in 76 limbs. PeriFlux LD was abnormal in 72 (94.7%) cases, SysToe in 69 (90.8%) cases, and MESI TBI in 70 (92.1%) cases.

MESI ABI combined with TBI measurement recognised 73 of 79 limbs (92.4%), thereby 42 of 44 (95.5%) PAD patients. If an automatic ABI cut-off level of 1.0 was taken, the number of recognised limbs rose to 74 (93.7%) and thus, 43 of 44 (97.7%) of the PAD patients could be recognised by the device.

## 4. Discussion

Our study aimed to evaluate how the automated, four-limb blood pressure monitor MESI mTablet could be applied for diagnostic and, especially, screening purposes of PAD. The measurement of the ABI works based on the oscillometric principle. An important additional function of the device is the assessment of toe pressure, thereby measuring the TBI using the PPG method.

The device works in a user-friendly way, and the measurements are easy to perform and do not require a considerable learning curve. In one study with the MESI ABPI MD device (a previous development of MESI Ltd., which measured blood pressure on three extremities simultaneously), no interobserver variability was found between the ABI readings obtained by medical students and experienced vascular specialists [39].

A noteworthy disadvantage of the device is that it does not perform exact numerical ABI measurements in case of incompressible arteries or severe PAD. As described in the user manual, at an ABI value “around or lower 0.5”, the text message of “abnormally weak pulse” is displayed. An analysis of the recorded oscillation graphs and pulse waveforms provides valuable additional information about the possibility of the forementioned two conditions; however, it also requires expertise on the examiner’s part.

The device provided a text message in about 10% of our ABI measurements. However, this draws attention to a limitation of our study, i.e., it involved a relatively small number of PAD patients. The ratio of measurements lacking numerical ABI values was about two times higher in diabetic patients. In our opinion, the fact that an exact ABI cannot be achieved in case of severe PAD limits the use of the device and does not allow a precise condition assessment of severe PAD patients and post-interventional follow-up. Due to these restrictions, our study aimed to focus primarily on the screening purposes of PAD. This is also supported by the fact that in all cases lacking numerical data, severe PAD lesions were detected using the vascular imaging techniques. Analysing the mean MESI ABI values of the different subgroups also emphasises the importance of PAD screening using the automated device because the ABI values of the control group differed significantly from the values of all other subgroups.

To our knowledge, two studies have been published so far comparing oscillometric measurements using the MESI ABPI MD device and the traditional Doppler method. Špan et al. found no considerable intrapatient differences with repeated measurements using the MESI device. The small coefficient of variation revealed excellent repeatability. In this study, a strong correlation (r = 0.61) was found between the ABI values obtained using the Doppler method and the MESI device. The Bland–Altman plot showed a mean difference of 0.06 ± 0.14, indicating an overestimation of pressure by the oscillometric device [40]. Our results, including a correlation coefficient of r = 0.67 and a mean ABI difference of 0.068 ± 0.15 in favour of the oscillometric method, are in good agreement with these findings. Similar to that study, we also detected proportional bias based on the comparison of the two methods with an overestimation of the lower and an underestimation of the higher ABI values.

These data also agree with the conclusions of a 2012 meta-analysis, where the average correlation between the Doppler and oscillometric ABI values was reported to be 0.71 ± 0.05. An average ABI difference between the oscillometric and Doppler ABI values of 0.020 ± 0.018 also suggested that the oscillometric measurement resulted in slightly higher ABI measurements [19].

In contrast to that, a recently published study reported poor measurement properties of the MESI ABPI MD device, obtaining numerical ABI results in only 63.4% of the ABI readings, thus resulting in a moderate correlation (r = 0.552) with the Doppler method. This study was performed in a patient cohort where 52% of all subjects was already diagnosed with PAD. A tendency to slightly overestimate the lower and underestimate the higher ABI values was also described. However, the mean inter-measurement difference of −0.067 ± 0.23 of the oscillometric and Doppler ABI indicated that the automated ABI readings resulted in lower average ABI values. Overall, the authors of this study suggested that this device was more sensitive in recognising already diagnosed PAD cases and less sensitive in establishing new diagnosis of PAD; therefore, the use of this device in primary care would not be recommended [41].

Similar to many other studies, both of these studies established the sensitivity and specificity values of oscillometric devices when compared to the Doppler method, where an ABI ≤ 0.9 indicated the diagnosis of PAD. We aimed to relate the automated ABI values to the results of the vascular imaging techniques, where the presence of an at least 50% stenosis of the lower limb arteries was defined as PAD. A small number of other studies that also took imaging techniques as the reference drew the conclusion that the generally accepted ABI cut-off value of 0.9 should be raised to increase the sensitivity and specificity of automated devices. An oscillometric ABI cut-off level of 0.95 was found to be optimal by Guo et al. to detect an at least 50% stenosis based on DSA [42]. Ichihashi et al. suggested the use of a cut-off level of 0.99 to detect an at least 50% stenosis based on CT angiography to achieve a sensitivity of 90% and a specificity of 85% [43]. Clairotte et al. recommended an oscillometric ABI cut-off value of 1.02 for unselected, 1.00 for non-diabetic and 1.04 for diabetic patients based on results of duplex ultrasound [44].

We found that the sensitivity of the MESI device in detecting an at least 50% lower limb stenosis could be increased from 57.0% to 74.7% by raising the automated ABI cut-off level from 0.9 to 1.0, without a significant decrease in specificity. The calculated optimal cut-off value of 0.99 would also make the clinical use of the automated ABI cut-off level “1.0” reasonable.

The sensitivity and specificity values obtained on the basis of our data support, on the one hand, the importance of calculating not only the standard but also the modified Doppler ABI values, and thus, a considerable increase in the sensitivity level (from 67.1% to 82.3%) could be achieved. The use of modified Doppler ABI values for more appropriate PAD diagnostics was also supported by other studies [45,46,47].

In diabetic patients, MESI had a comparable diagnostic efficacy with the traditional Doppler method (sensitivity of 53.7% for the former and 56.1% for the latter). These moderate sensitivity values emphasise the importance of toe pressure measurement in detecting PAD. Since toe arteries are usually not affected by MAC, the measurement of systolic toe pressure helps overcome the limitations resulting from falsely elevated ankle pressure values due to mediasclerosis [20,21,24]. The two most common measurement methods of toe pressure, LD flowmetry and photophlethysmography, have been validated in some studies and are considered a reliable alternative for each other [26,48,49,50,51,52].

Although some portable, battery-powered, partly or fully automated devices are already available, their widespread use in primary care has not been realised due to cost or personnel factors. Unfortunately, even among medical personnel, the importance of toe pressure measurement is not emphasised enough. Despite the fact that it can eliminate the limitations of ABI measurement, for PAD screening, TBI is almost never measured routinely. A meta-analysis published in 2020 concluded that the measurement of TBI is more sensitive [81% (95% CI: 70–94)] than the measurement of ABI [61% (95% CI: 55–69)] at the cost of lower specificity [92% (95% CI: 89–95) for ABI and 77% (95% CI: 66–90) for TBI]. Moreover, partly because it can also diagnose PAD in the case of MAC, this meta-analysis considered it a better screening tool than the measurement of ABI [53]. However, only a few studies examining the screening possibilities of PAD included the combined examination of ABI and TBI [54,55,56,57,58], and most of them involved only patients with diabetes [54,55,56,57]. Since the MESI mTablet unites the ABI and TBI measurements in one, easily operated device, the possibility of sequential measurements could facilitate more widespread use. Based on our data, no significant differences could be found between the three types of measurement techniques (PeriFlux LD, SysToe and MESI PPG measurements, respectively). The Bland–Altman analysis revealed good intermodality agreement with the results of LD flowmetry, and the ROC curves showed an excellent diagnostic efficiency for all three methods. However, two pitfalls of the automatic MESI TBI measurement should be noted. On the one hand, the measurement range covers 20 to 250 mmHg, so in contrast to the LD fluxmetry, no accurate measurement can be carried out in very low range of toe pressures, which affected 4.8% of our measurements. It should also be noted that an operator cannot completely rely on automated toe pressure measurement since the perfusion curve has to be manually adjusted in most cases due to movement artifacts. Despite these disadvantages, toe pressure measurement provides a very valuable addition to ABI measurement. When the standard Doppler and MESI ABI readings were compared for screening purposes, MESI was underpowered compared to the Doppler method (57.0% vs. 70.9% of all limbs affected by PAD as recognised by MESI and Doppler ABI). In our sample, the two methods performed similarly in diabetic patients, and the differences resulted mainly from the measurements of non-diabetic patients. If MESI ABI was combined with TBI measurement, the proportion of limbs recognised as pathological rose to 92.4%. The moderate sensitivity of MESI ABI readings could be substantially improved by taking an automated ABI cut-off level of 1.0. If measurement using the TBI module was additionally performed, an excellent sensitivity of 93.7% could be achieved.

### Study Limitations

Our study involved a relatively small number of previously confirmed PAD patients. Measurements, including colour-coded duplex ultrasound examination, were performed by one independent operator. The generally subjective evaluation, the difficulties in assessment of lesions of the iliac arteries and on the calf using vascular ultrasound examination, and the use of three different vascular imaging techniques might have also limited the investigation.

## 5. Conclusions

In summary, despite the drawbacks resulting from the technical specifications of the MESI mTablet device, our data suggest that if an ABI cut-off level of 1.0 is used and a sequential TBI measurement is performed, the device could be efficiently applied for screening purposes. The easy and user-friendly implementation of the measurements may contribute to its widespread use in primary care or in screening programmes; however, the precise interpretation of the obtained results requires some expertise.

## Figures and Tables

**Table 1:** Demographics and baseline characteristics of the study sample representing medical history, co-morbidities, cardiovascular risk factors, concomitant medication and lower limb symptoms of patients belonging to various subgroups and of all patients. (SD, standard deviation; BMI, body mass index; DOAC, direct oral anticoagulant; ACEI, angiotensin-converting enzyme inhibitor; ARB, angiotensin receptor blocker; CV, cardiovascular; PAD, peripheral artery disease).

	Control	Other CV	High CV Risk	Very High CV Risk	Confirmed PAD	All Patients
	(*n* = 18)	(*n* = 12)	(*n* = 28)	(*n* = 28)	(*n* = 31)	(*n* = 117)
Age (mean ± SD, years)	60.4 ± 11.9	42.7± 11.9	61.6 ± 10.6	69.1 ± 10.0	68.8 ± 7.9	63.2 ± 12.8
Male sex No (%)	9 (50.0%)	5 (41.7%)	7 (25.0%)	10 (35.7%)	18 (58.1%)	49 (41.9%)
BMI (mean ± SD, kg/m^2^)	24.6 ± 3.6	31.5 ± 11.3	32.9 ± 7.5	30.9 ± 6.9	27.7 ± 6.6	29.6 ± 7.6
Co-morbidities and risk factors No (%)						
Hypertension	6 (33.3%)	2 (16.7%)	27 (96.4%)	27 (96.4%)	29 (93.5%)	
Diabetes mellitus	0 (0%)	0 (0%)	17 (60.7%)	8 (28.6%)	16 (51.6%)	
Diabetic polyneuropathy	0 (0%)	0 (0%)	5 (17.9%)	4 (14.3%)	5 (16.1%)	
Dyslipidaemia	4 (22.2%)	0 (0%)	18 (64.3%)	25 (89.3%)	28 (90.3%)	
Smoker (current)	0 (0%)	3 (25.0%)	7 (25.0%)	7 (25.0%)	11 (35.5%)	
Smoker (former)	4 (22.2%)	2 (16.7%)	7 25.0%)	7 (25.0%)	16 (51.6%)	
Coronary heart disease	0 (0%)	0 (0%)	0 (0%)	13 (46.4%)	8 (25.8%)	
Carotid artery disease	0 (0%)	0 (0%)	0 (0%)	8 (28.6%)	7 (22.6%)	
Cerebrovascular events	0 (0%)	0 (0%)	0 (0%)	6 (21.4%)	5 (16.1%)	
Renal artery disease	0 (0%)	0 (0%)	0 (0%)	0 (0%)	0 (0%)	
Chronic kidney disease	0 (0%)	0 (0%)	0 (0%)	2 (7.1%)	3 (9.7%)	
Concomitant medication No (%)						
Aspirin	0 (0%)	2 (16.7%)	8 (28.6%)	7 (25.0%)	11 (35.5%)	
Clopidogrel	0 (0%)	0 (0%)	1 (3.6%)	6 (21.4%)	20 (64.5%)	
Cilostazol	0 (0%)	0 (0%)	0 (0%)	1 (3.6%)	7 (22.6%)	
Oral anticoagulants						
Vitamin K antagonist	0 (0%)	0 (0%)	0 (0%)	3 (10.7%)	0 (0%)	
DOAC	0 (0%)	4 (19.0%)	6 (21.4%)	9 (32.1%)	6 (19.4%)	
ACEI/ARB	6 (33.3%)	2 (16.7%)	24 (85.7%)	20 (71.4%)	24 (77.4%)	
Beta blocker	4 (22.2%)	5 (41.7%)	12 (42.9%)	16 (57.1%)	21 (67.7%)	
Calcium channel blocker	2 (11.1%)	2 (16.7%)	14 (50.0%)	10 (35.7%)	14 (45.2%)	
Statin	3 (16.7%)	0 (0%)	10 ((35.7%)	20 (71.4%)	25 (80.1%)	
Fibrate	0 (0%)	0 (0%)	2 (7.1%)	1 (3.6%)	0 (0%)	
Ezetimibe	0 (0%)	0 (0%)	1 (3.6%)	3 (10.7%)	3 (9.7%)	
Lower limb symptoms No (%)						
Leg ulcers	0 (0%)	0 (0%)	0 (0%)	1 (3.6%)	5 (16.1%)	
Ischaemic rest pain	0 (0%)	0 (0%)	0 (0%)	0 (0%)	5 (16.1%)	
Intermittent claudication	0 (0%)	1 (8.3%)	2 (7.1%)	6 (21.4%)	17 (54.8%)	

**Table 2:** ABI values obtained using the Doppler, modified Doppler and MESI methods in the various subgroups of patients (ABI, ankle–brachial index; CV, cardiovascular; PAD, peripheral artery disease).

	Control (36 Limbs)	Other CV (24 Limbs)	High CV Risk (56 Limbs)	Very High CV Risk (56 Limbs)	Confirmed PAD (61 Limbs)
Doppler ABI	1.158 ± 0.091	1.109 ± 0.133	1.093 ± 0.164	1.006 ± 0.232	0.749 ± 2.262
+incompressible arteries (*n* = limbs)	0 (0.0%)	0 (0.0%)	0 (0.0%)	5 (8.9%)	1 (1.6%)
Doppler ABI modified	1.096 ± 0.864	1.051 ± 0.137	1.007 ± 0.139	0.947 ± 0.228	0.572 ± 0.320
+incompressible arteries (*n* = limbs)	0 (0.0%)	0 (0.0%)	0 (0.0%)	3 (5.4%)	0 (0.0%)
MESI ABI	1.230 ± 0.659	1.144 ± 0.097	1.273 ± 0.101	1.115 ± 0.100	0.945 ± 0.183
+only text data (*n* = limbs)	0 (0.0%)	0 (0.0%)	1 (1.8%)	3 (5.4%)	19 (31.1%)

**Table 3:** TBI values obtained using PeriFlux LD, SysToe and MESI in the various subgroups of patients (LD, laser Doppler; TBI, toe–brachial index; CV, cardiovascular; PAD, peripheral artery disease).

	Control (36 Toes)	Other CV (24 Toes)	High CV Risk (56 Toes)	Very High CV Risk (56 Toes)	Confirmed PAD (58 Toes)
PeriFlux LD TBI	0.822 ± 0.068	0.801 ± 0.206	0.751 ± 0.193	0.698 ± 0.189	0.399 ± 0.181
SysToe TBI	0.846 ± 0.076	0.781 ± 0.189	0.768 ± 0.188	0.708 ± 0.176	0.439 ± 0.198
MESI TBI	0.838 ± 0.125	0.765 ± 0.347	0.766 ± 0.211	0.703 ± 0.252	0.344 ± 0.239

## Data Availability

The datasets that support the findings of this study are available from the corresponding author (K. F.) upon reasonable request. The data are not publicly available due to ethical and privacy issues.
